# HNCcorr: A Novel Combinatorial Approach for Cell Identification in Calcium-Imaging Movies

**DOI:** 10.1523/ENEURO.0304-18.2019

**Published:** 2019-04-15

**Authors:** Quico Spaen, Roberto Asín-Achá, Selmaan N. Chettih, Matthias Minderer, Christopher Harvey, Dorit S. Hochbaum

**Affiliations:** 1 Department of Industrial Engineering & Operations Research, University of California, Berkeley, CA 94720-2284; 2 Department of Computer Science, Universidad de Concepción, Concepción, Chile; 3 Department of Neurobiology, Harvard Medical School, Boston, MA 02115

**Keywords:** calcium imaging, cell identification, clustering, combinatorial optimization, graph methods

## Abstract

Calcium imaging is a key method in neuroscience for investigating patterns of neuronal activity *in vivo*. Still, existing algorithms to detect and extract activity signals from calcium-imaging movies have major shortcomings. We introduce the HNCcorr algorithm for cell identification in calcium-imaging datasets that addresses these shortcomings. HNCcorr relies on the combinatorial clustering problem HNC (Hochbaum’s Normalized Cut), which is similar to the Normalized Cut problem of Shi and Malik, a well known problem in image segmentation. HNC identifies cells as coherent clusters of pixels that are highly distinct from the remaining pixels. HNCcorr guarantees a globally optimal solution to the underlying optimization problem as well as minimal dependence on initialization techniques. HNCcorr also uses a new method, called “similarity squared”, for measuring similarity between pixels in calcium-imaging movies. The effectiveness of HNCcorr is demonstrated by its top performance on the Neurofinder cell identification benchmark. We believe HNCcorr is an important addition to the toolbox for analysis of calcium-imaging movies.

## Significance Statement

Calcium imaging is a method for recording neuronal activity at a cellular resolution that requires automated approaches to identify cells and their signals. HNCcorr is a novel algorithm that identifies these cells successfully and efficiently. HNCcorr is unique in that it addresses an optimization model and delivers a guaranteed globally optimal solution, thus ensuring a fully transparent link between the input data and the resulting cell identification. This contrasts with existing state-of-the-art approaches that produce only heuristic solutions to the underlying optimization model, and consequently may miss cells due to the suboptimality of the generated solutions.

## Introduction

Calcium imaging has become a standard method to measure neuronal activity *in vivo* (Stosiek et al., 2003). Using genetically encoded calcium indicators and fast laser-scanning microscopes, it is now possible to record thousands of neurons simultaneously. However, the manual postprocessing needed to extract the activity of single neurons requires tens of hours per dataset. Consequently, there is a great need for automated approaches for the extraction of neuronal activity from imaging movies.

There are three classes of existing techniques for cell identification in calcium-imaging movies: semi-manual region of interest (ROI) detection (Kaifosh et al., 2014; Driscoll et al., 2017), shape-based detection algorithms [Pachitariu et al., 2013; Apthorpe et al., 2016; Klibisz et al., 2017; S. Gao, (https://bit.ly/2UG7NEs)], and matrix factorization algorithms (Mukamel et al., 2009; Pnevmatikakis and Paninski, 2013; Pnevmatikakis et al., 2013a, 2016; ; Diego-Andilla and Hamprecht, 2014; Maruyama et al., 2014; Pachitariu et al., 2016; Levin-Schwartz et al., 2017). Semi-manual ROI detection techniques rely on the user’s input for detecting and segmenting cells. This process has been reported to be highly labor intensive (Resendez et al., 2016) and may miss cells with a low signal-to-noise ratio or a low activation frequency. Shape-based identification methods locate the characteristic shapes of cells using deep learning [Apthorpe et al., 2016; Klibisz et al., 2017; S. Gao, (https://bit.ly/2UG7NEs)] or dictionary learning (Pachitariu et al., 2013). Shape-based techniques are typically applied by compressing the movie into a summary image obtained by averaging over the time dimension. The third class of techniques uses a matrix factorization model to decompose a movie into the spatial and temporal properties of the individual neuronal signals. The matrix factorization algorithm CNMF (Pnevmatikakis et al., 2016) is currently prevalent for the task of cell identification.

We propose here a vastly different approach, called HNCcorr, based on combinatorial optimization. The cell identification problem is formalized as an image segmentation problem where cells are clusters of pixels in the movie. To cluster the cells, we use the clustering problem Hochbaum’s Normalized Cut (HNC) (Hochbaum, 2010, 2013). This problem is represented as a graph problem, where nodes in the graph correspond to pixels, edge weights correspond to similarities between pairs of pixels, and an objective function assigns a cost to any possible segmentation of the graph. The objective function used in HNC provides a trade-off between two criteria: one criterion is to maximize the total similarity of the pixels within the cluster, whereas the second criterion is to minimize the similarity between the cluster and its complement. Highly efficient solvers exist to solve HNC optimally (Hochbaum, 2010, 2013).

The name HNCcorr is derived from two major components of the algorithm: the clustering problem HNC (Hochbaum, 2010, 2013), and the use of a novel similarity measure derived from correlation, named (sim) ^2^ for “similarity squared”.

The idea of (sim) ^2^ is to associate with each pixel a feature vector of correlations with respect to a subset of pixels, and to determine the similarities between pairs of pixels by computing the similarity of the respective two feature vectors. An important feature of (sim) ^2^ over regular pairwise correlation is that it considers any two background pixels, pixels not belonging to a cell, as highly similar, whereas correlation deems them dissimilar. This improves the clustering since it incentivizes that background pixels are grouped together.

An advantage of HNCcorr compared with most alternative algorithms is that the HNC optimization model used to identify cells is solved efficiently to global optimality. This makes the output of the optimization model transparent in the sense that the effect of the model input and parameters on the resulting optimal solution is well understood. In contrast, most other approaches, such as matrix factorization algorithms, rely on intractable optimization models. This means that the algorithms cannot find a global optimal solution to their optimization model. Instead, they find a locally optimal solution close to the initial solution. As a result, the algorithms provide no guarantee on the quality of the delivered solutions and cells may remain undetected. See Discussion for more details.

The experimental performance of the HNCcorr is demonstrated on the Neurofinder benchmark (CodeNeuro, 2016) for cell identification in annotated two-photon calcium-imaging datasets. This benchmark is currently the only available benchmark that objectively evaluates cell identification algorithms. On this benchmark, HNCcorr achieves a higher average F1-score than two frequently used matrix factorization algorithms CNMF (Pnevmatikakis et al., 2016) and Suite2P (Pachitariu et al., 2016).

We further provide a comparison between HNCcorr and a procedure based on spectral clustering in which we demonstrate that HNCcorr attains a higher F1-score. We also present a running time comparison among the MATLAB implementations of HNCcorr, CNMF, and Suite2P. HNCcorr has similar running time performance as Suite2P and is approximately 1.5 times faster than CNMF.

A MATLAB implementation of HNCcorr is available at https://github.com/quic0/HNCcorr. A Python implementation of HNCcorr is forthcoming.

## Materials and Methods

### The HNCcorr algorithm

The HNCcorr algorithm addresses the problem of cell identification in calcium-imaging datasets. The goal is to identify fluorescence sources, such as neuronal cell bodies. HNCcorr aims to find active cells that are characterized by a distinct time-varying signal. HNCcorr finds a single cell by solving a clustering problem called HNC. The solution is a cluster of pixels that are highly similar to each other but distinct from the pixels not in the cluster.

The output of the HNCcorr algorithm is the spatial footprint for each detected cell in a motion-corrected movie. The spatial footprint is a set of pixels that represents the location of the cell in the imaging plane of the movie. These footprints are used for signal extraction (e.g., by averaging the intensity of the pixels in the footprint and subtracting an estimated background signal or with the use of more advanced algorithms; Vogelstein et al., 2010; Grewe et al., 2010; Pnevmatikakis et al., 2013b; Theis et al., 2016; Jewell and Witten, 2018).

The HNCcorr algorithm identifies all cells in the dataset by processing a set of positive seeds. A positive seed represents a potential location of a cell. For each positive seed, the algorithm repeats the following three phases: (1) *input preparation*: construct the similarity graph as the input for the HNC clustering problem; (2) *HNC clustering*: optimally solve the HNC clustering model on the similarity graph; and (3) *postprocessing*: evaluate the output produced by HNC to decide whether a cell was detected and to identify the footprint of a cell.

We now describe each part in detail. We first discuss how the input for the HNC clustering problem is prepared. Subsequently, we define the HNC clustering problem, the role of each of the model inputs, and the solution technique used to solve the problem. To conclude, we describe how the output produced by the HNC problem is postprocessed.

### Input preparation

The input for the HNC clustering problem consists of the seeds and the similarity graph with the corresponding similarity weights. Below we describe how these inputs are constructed.

#### Seed selection

The positive seeds each indicate a potential location of a cell in the dataset. They are generated in advance and processed one at a time. With each positive seed the goal is to identify a new cell. The positive seed indicates the cell of interest. To ensure that this potential cell is segmented, the positive seed must be contained in the candidate clusters returned by the HNC problem.

A positive seed is a superpixel, a square of *k* × *k* pixels in two-dimensional datasets. *k* is typically set to 3 or determined by validation on an annotated dataset. The set of positive seeds is generated by a seed selection procedure. By default, HNCcorr uses a procedure that selects pixels with the highest average correlation to their neighboring pixels. It is described in detail in the algorithmic implementation section. Alternatively, it is also possible to enumerate all superpixels of a given size.

In addition to the positive seed, the HNC problem also requires a set of negative seeds. These negative seeds are pixels that cannot be selected as part of the cluster in the HNC problem. The use of negative seeds ensures that not all pixels are selected. The negative seeds are selected uniformly from a circle centered at the positive seed. The radius of the circle is chosen such that the any cell that contains the positive seed is inside the circle. See Figure 3*B* for an example of both the positive seeds and the negative seeds.

#### Generating patches

For each positive seed, we limit the data that is considered to a square of pixels centered at the positive seed. This square of pixels is referred to as a “patch.” It is large enough to ensure that any cell is fully contained in the patch. Its size is thus dependent on the spatial resolution of the data. Note that patches that correspond to different positive seeds may overlap.

#### Graph construction

For a given patch with positive and negative seeds, we construct a graph *G* = (*V*, *E*) consisting of a set of nodes, denoted by *V*, and a set of edges that connect pairs of nodes, denoted by *E*. The set of nodes *V* is the set of pixels in the patch. The construction of the edge set *E* is described after we discuss the similarity weights associated with each edge.

#### Similarity weights

Every edge [i,j]∈E has an associated similarity weight $w_{ij}\in [0,1]$. This similarity weight *w_ij_* measures the similarity between pixels *i* and *j* with higher values indicating increased similarity between the two pixels.

A common approach to assess the similarity between two pixels is to measure the correlation between their fluorescence signals across all frames of the movie. To express this mathematically, we define $x_{u}\in {\mathbb{R}}^{T}$ as the fluorescence vector of pixel u∈V for a movie consisting of *T* frames. The correlation corr(u,v) between pixels *u* and *v* is defined as follows:

$$\text{corr}(u,v)=\frac{1}{T-1}\frac{{\left(x_{u}-\hat{\mu }(x_{u})\right)}^{T}(x_{v}-\hat{\mu }(x_{v}))}{\hat{\sigma }(x_{u})\;\hat{\sigma }(x_{v})},$$

where $\hat{\mu }(x)$ and $\hat{\sigma }(x)$ are, respectively, the sample mean and sample standard deviation of the vector **x**.

Correlation effectively measures the similarity between two pixels from the same cell, since such pixels have correlated fluorescence patterns due to the shared signal of the cell. For another important group of pixels, the background pixels, correlation is less effective. The background pixels are the pixels that do not belong to a cell. As such, background pixels are at best weakly correlated with each other and with other pixels. Yet the lack of strong correlation with other pixels is effectively what characterizes background pixels.

We can use this observation to strengthen the similarity measure and to aid the clustering. In other words, the background pixels are similar to each other in the pattern of being weakly correlated to every other pixel, whereas pixels that belong to a cell are highly correlated to other pixels in the same cell. Instead of computing the similarity between pixels directly based on their correlation, we compare how pixels correlate with all pixels.

**Figure 1.**
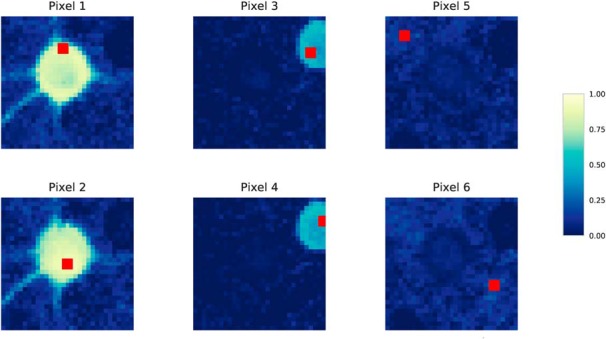
Visualization of the correlation images of six pixels. The correlation image is a two-dimensional visualization of the feature vector *R_i_* of pixel *i* (e.g. *R*_1_ for pixel 1). The color (value) of each pixel shown in each correlation image is the pixel-to-pixel correlation between that pixel and the pixel marked in red. Lighter colors represent higher correlations, with the correlation scale truncated at zero. The patch is taken from the Neurofinder 02.00 training dataset and contains two cells. Pixels 1 and 2 belong to the same cell, pixels 3 and 4 belong to the same cell, and pixels 5 and 6 are background pixels. Although the pairs of pixels 1 and 2, pixels 3 and 4, and pixels 5 and 6 are not always highly correlated, their correlation images are nearly identical.

Figure 1 illustrates this. For a small patch, six pixels are marked in red and are shown with their pairwise correlation to all pixels in the patch. We refer to these images as correlation images. Two pixels in each of the two visible cells are marked as well as two background pixels. The correlation image for each pixel (e.g., pixel 1) shows the pairwise correlation between pixel 1 and every pixel in the patch, represented by the color of the pixel, with a lighter color corresponding to higher correlation. The following observations are drawn from the figure: pixels belonging to the same cell have nearly identical correlation images (see pixels 1 and 2 and pixels 3 and 4). Furthermore, background pixels also have nearly identical correlation images, although the pixels themselves are not correlated (see pixels 5 and 6).

Similarity between background pixels can thus be captured by comparing their correlation images, whereas correlation or other standard similarity measures would not recognize this. Comparing correlation images instead of computing signal correlation also boosts the similarity between pixels in cells with a low signal-to-noise ratio, such as the cell containing pixels 3 and 4. Although pixels 3 and 4 are only weakly correlated, their correlation images are nearly identical.

This approach is an application of a novel technique for computing pairwise similarities that we call (sim) ^2^. The idea of (sim) ^2^ is to determine the similarity between a pair of objects (here pixels), not by directly comparing their features, but rather by comparing the similarities of the objects to a set of reference objects, called the “reference set”. The resulting pairwise similarities between objects can be interpreted as a similarity derived from other similarities, hence the name (sim) ^2^.

Our use of (sim) ^2^ here consists of the following two-step procedure: in the first step, HNCcorr evaluates the pairwise correlation between each pixel and all pixels in the patch. The resulting vector of correlations *R_i_* is the feature vector of pixel *i*. Assuming that there are *n* pixels in the patch, *R_i_* is a vector of dimension *n*. Mathematically, the *k*^th^ element of the feature vector *R_i_* of pixel i∈V is defined as $R_{i}[k]=\text{corr}(i,k)$. Alternatively, the feature vector can be interpreted as a vector representation of the correlation image of pixel *i* or as the row of pixel *i* in the pixel-to-pixel correlation matrix defined over the pixels in the patch.

In the second step, the similarity weights *w_ij_* are computed for every edge [i,j]∈E as the Gaussian similarity between the feature vectors, as follows:

(1) $$w_{ij}=\exp(-\alpha ∥R_{i}-R_{j}∥{}_{2}^{2}),$$

where *α* is a scaling parameter, which is typically set to 1. When the correlation images of pixels *i* and *j* are identical, then *w_ij_* = 1. *w_ij_* approaches zero when the correlation images are highly dissimilar. The proposed method is independent from the length of the movie except for the calculation of the correlation coefficients.

#### Selecting relevant similarities with sparse computation

**Figure 2.**
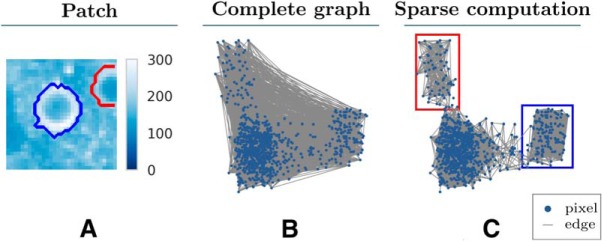
Sparse computation constructs a sparse similarity graph. Comparison of a complete similarity graph and the similarity graph constructed by sparse computation for an example patch. For the purpose of illustration, the nodes are positioned based on the two-dimensional PCA projection of the feature vectors of pixels offset by a small uniformly sampled perturbation. ***A***, Mean intensity image of the patch with the outline of two cells marked in red and blue. ***B***, Complete similarity graph with an edge between every pair of pixels. For the purpose of illustration, only 10,000 randomly sampled edges are shown. ***C***, Sparse similarity graph constructed by sparse computation with a three-dimensional PCA projection and a grid resolution of *κ* = 25. Two clusters of pixels (marked with red and blue rectangles) are identified by Sparse Computation. These two clusters match the spatial footprints of the two cells shown in ***A***.

A naive approach for selecting the edge set *E* is to connect every pair of pixels. Instead, HNCcorr relies on a technique called sparse computation (Hochbaum and Baumann, 2015; Baumann et al., 2016) that recognizes the relevant pairwise similarities without first computing all pairwise similarities. Sparse computation effectively removes similarities that it recognizes as negligible without computing them first. For each pairwise similarity identified as negligible, there is no edge connecting the respective pair, rendering the graph sparse. An example of the effect of sparse computation is shown in Figure 2.

The use of sparse computation provides various advantages, such as improving the running time. For general machine-learning problems, sparse computation significantly reduces the running time of a similarity-based classifier at almost no loss in accuracy (Hochbaum and Baumann, 2015; Baumann et al., 2016). For HNCcorr, we observe that sparse computation improves the running time by at least one order of magnitude compared with using a complete similarity graph.

The sparse computation method considers each pixel i∈V as an object represented by its feature vector *R_i_*, as defined previously. Sparse computation first projects these feature vectors onto a low-dimensional space of dimension *p* using a fast approximation of principal component analysis (PCA) (Drineas et al., 2006; Hochbaum and Baumann, 2015). The low-dimensional space is then subdivided into a grid with *κ* sections per dimension, resulting in a total of *κ^p^* grid blocks. Pairs of pixels are considered relevant similarities if the pixels fall in the same or adjacent grid blocks in the low-dimensional space. These pairs of objects are selected for the edge set *E*. For these pairs, we will compute the pairwise similarities.

### The HNC clustering model

The optimization model used to identify the footprint of a single cell is the HNC model (Hochbaum, 2010, 2013). HNC is closely related to a well-known optimization problem called the Normalized Cut, from the field of image segmentation (Shi and Malik, 2000). The input to HNC is a patch, the corresponding graph *G* = (*V*,*E*), and seeds. The HNC model is defined for a partition of the pixels into the sets *S* and $\overset{¯}{S}=V\,\;\backslash \;S$. The cluster *S* will represent the spatial footprint of a cell and must contain the positive seed. The set $\overset{¯}{S}=V\;\backslash \;S$ consists of the remaining pixels and must contain the negative seeds.

The HNC problem aims to find a cluster *S* such that is coherent and distinct from $\bar{S}$. Distinctness is attained by reducing the similarity between pixels in *S* and $\bar{S}$. Coherence is achieved by maximizing the similarity between the pixels in the set *S*. The HNC problem trades off these two objectives as follows:

(*λ*-HNC) $$\underset{\emptyset \subset S\subset V}{\min}\overset{\text{Distinctness of }S}{\overset{︷}{\underset{\begin{array}{c} {}[i,j]\in E,\\ i\in S,\;j\in \overset{¯}{S} \end{array}}{\sum }w_{ij}}}-\lambda \overset{\text{Coherence of}\;S}{\overset{︷}{\underset{\begin{array}{c} {}[i,j]\in E,\\ i\in S,\;j\in S \end{array}}{\sum }w_{ij}}}.$$

The relative weight of the two objectives is determined by λ≥0. S^*^(*λ*) is used to denote the optimal cluster for each value of *λ*. The algorithm for solving HNC simultaneously determines the optimal solutions for all possible values of *λ*, as explained below.

While the HNC problem is solved efficiently, the Normalized Cut problem is an NP-Hard problem (Shi and Malik, 2000). This implies that it is extremely unlikely that an efficient algorithm exists that optimally solve the Normalized Cut problem. Instead, spectral clustering is often used as a heuristic. Although spectral clustering is a popular method, Hochbaum et al. (2013) and Hochbaum (2013) demonstrates that HNC dominates spectral clustering in terms of visual quality and practical efficiency for a set of benchmark images taken from the Berkeley Segmentation Dataset (Martin et al., 2001).

The algorithm to solve the HNC problem can find the optimal solutions for all values of *λ* simultaneously (Hochbaum, 2010, 2013), removing the need for tuning the *λ* parameter. This algorithm utilizes parametric minimum cut/maximum flow algorithms (Gallo et al., 1989; Hochbaum, 2008). An intuitive explanation of why it is possible to find the solutions for all values of *λ* is that the sets *S* ^*^(*λ*) were shown to be nested (Goldberg and Tarjan, 1988; Hochbaum, 2008, 2010). That is, if $\lambda_{1}<\lambda_{2}$ then $S^{*}(\lambda_{1})\subseteq S^{*}(\lambda_{2})$. Therefore, there are at most n=|V| such distinct sets, where *n* is the number of pixels in the patch. Each of these nested sets $S_{1}^{*}\subset S_{2}^{*}\subset \ldots \subset S_{\ell }^{*}$ has an associated *λ*-interval $\left[\lambda_{i},\lambda_{i+1}\right)$ for which the set is optimal. These sets form the output of the HNC algorithm and serve as candidates for the footprint of the cell.

### Postprocessing

As output of the HNC problem, we obtain all nested optimal sets $S_{1}^{*}\subset S_{2}^{*}\subset \ldots S_{\ell }^{*}$. These clusters are candidates for the footprint of a cell. The postprocessing algorithm decides whether a cell was detected and identifies its spatial footprint based on the candidate clusters $S_{i}^{*}$ for i=1,…,ℓ.

In the current implementation, the postprocessing technique decides whether a cell was found based on the sizes of the candidate clusters. A cluster $S_{i}^{*}$ is discarded if the number of pixels in the cluster, $\left|S_{i}\right|$ falls outside a given size range. In case all clusters are discarded, then the algorithm concludes that no cell was detected. In case one or more candidates remain, each remaining cluster *S_i_* is compared to a preferred cell size. The candidate cluster that is closest in size to the expected cell size is selected as the spatial footprint of the cell. We use $\sqrt{\left|S_{i}\right|}$ for this comparison since this best reflects the scaling of the area of a circle. More complex postprocessing techniques based on convolutional neural networks have been explored as well. However, preliminary experiments showed no substantial improvements.

### Summary

**Figure 3.**
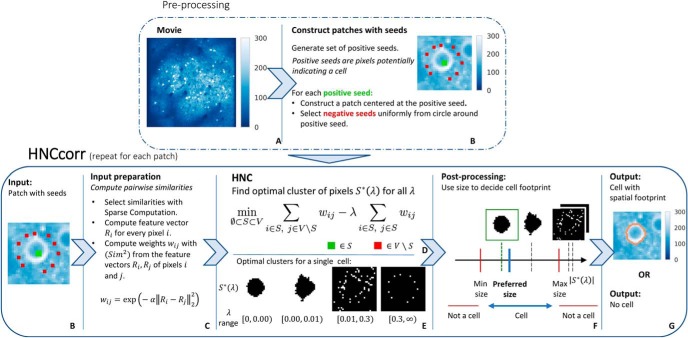
Overview of the HNCcorr algorithm. The top and bottom rows, respectively, summarize the preprocessing steps and the main steps of the algorithm. ***A***, Average intensity image of the input dataset consisting of a calcium-imaging recording. ***B***, Average intensity image of a patch constructed for a positive seed (green) and the corresponding negative seeds (red). ***C***, Description for computing the pairwise similarity weight between two pixels. ***D***, HNC is the clustering model solved to segment a single cell. ***E***, Optimal clusters for the HNC problem as a function of *λ*. Black pixels are selected for the cluster, denoted by *S* ^*^(*λ*). ***F***, Visualization of the postprocessing step. Clusters that are too small/large are discarded. The remaining cluster closest to the preferred size is selected as the footprint of a cell. ***G***, Output for a single patch; the footprint of a cell if a cluster was selected, or “No cell” if all clusters are discarded.

The main steps of the HNCcorr algorithm are summarized in Figure 3.

### Algorithmic implementation of HNCcorr

We provide a detailed algorithmic description of HNCcorr for reproducibility of the results. It discusses the implementation of (sim) ^2^, the main routine of HNCcorr for segmenting a single cell, the seed selection method, and the postprocessing of the segmentations. A MATLAB implementation is available on GitHub at https://github.com/quic0/HNCcorr. A Python implementation is forthcoming.

#### (sim)^2^ implementation

We extend here the description of how HNCcorr uses (sim) ^2^. Instead of computing the pairwise correlations with respect to all pixels in the patch, we compute only the pairwise correlation with respect to a sampled subset of pixels. That is, the reference set consists of a random subset of pixels in the patch. This change is made to reduce the running time of the feature vector computation. It has a negligible effect on the algorithmic performance if at least a fourth of the pixels are sampled for the reference set.

The implementation has the following three steps: first, we sample a subset of the pixels in the patch to form the reference set; second, we compute, for every pixel *i* in the patch, the feature vector *R_i_* consisting of the pairwise correlations to the pixels in the reference set; and third, we compute the Gaussian similarity between *R_i_* and *R_j_* for every edge [i,j]∈E. We now describe each step in more detail.

For the first step, recall that *V* is the set of pixels in a patch and let *RS* be the reference set. The reference set *RS* consists of pixels randomly sampled with replacement from *V*. If |V| denotes the number of pixels in the patch and *γ* is the sampling rate, then the reference set *RS* consists of pixels $r_{1},\ldots ,r_{k}$ for k=γ|V|.

In the second step, we compute the feature vectors $R_{i}\in {\mathbb{R}}^{\left|RS\right|}$ where |RS| is the size of the reference set. The *h*^th^ component, for h=1,…,k of the feature vector *R_i_* of pixel *i* is defined as follows:

$$R_{i}[h]=\text{corr}(i,r_{h}).$$

In the third step, the similarity weight associated with edge [i,j]∈E is computed. This weight is defined as follows:

$$w_{ij}=\exp(-\alpha ∥R_{i}-R_{j}∥{}_{2}^{2}),$$

where *α* is typically set to 1. Note that $w_{ij}\neq \text{corr}(i,j)$.

#### HNCcorr: single-cell segmentation

The implementation of how HNCcorr processes a single seed is described in algorithm 1.

**Algorithm 1:**
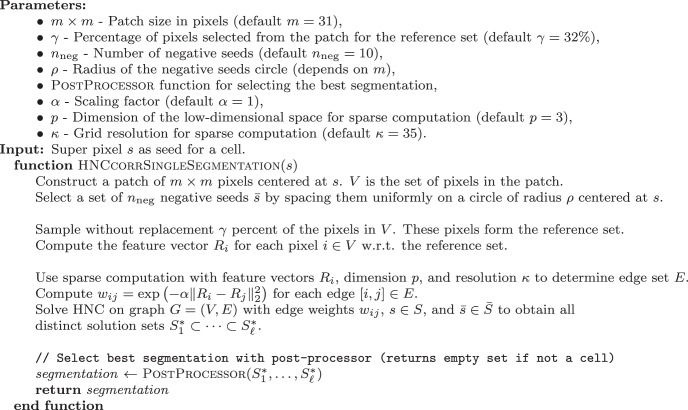
HNCcorr - Single cell segmentation.

The default value of *γ* = 32% was determined based on an experimental evaluation of the cell detection performance and the running time performance. The value of 32% provides a substantial improvement in running time compared to *γ* = 100% and almost no loss in cell detection accuracy.

#### Seed selection algorithm

The HNCcorr algorithm allows for any, possibly parallelized, seed selection procedure. The seed selection procedure (algorithm 2) used here is an enumeration algorithm with a few speedups. The algorithm first partitions the pixels into *n*_grid_ × *n*_grid_ grid blocks, where *n*_grid_ = 5 by default. Next, it selects one pixel per grid block with the highest average correlation to its eight neighboring pixels. These selected pixels are sorted from high to low in terms of average correlation to its neighborhood. The top *p*_seed_ percentage of pixels are selected as the centers of the positive seeds. Typically, *p*_seed_ is set to 40%. This value was decided based on an empirical study. This study indicates that the benefit of increasing it above 40% is small for most datasets, whereas it increases running time. Note that this method is equivalent to full enumeration if we partition the pixels into a grid blocks with a single pixel (*n*_grid_ = 1), and all selected pixels are kept (*p*_seed_ = 100%).

**Algorithm 2:**
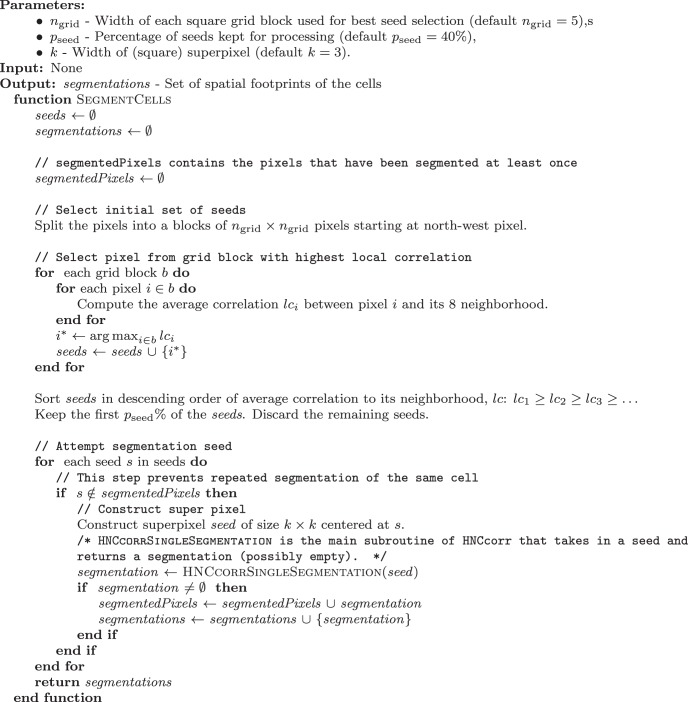
Seed selection and outer loop for HNCcorr algorithm.

#### Size-based postprocessor

The current implementation of HNCcorr postprocesses each seed and the corresponding segmentations based on the size of the segmentations. The implementation is defined in algorithm 3.

**Algorithm 3:**
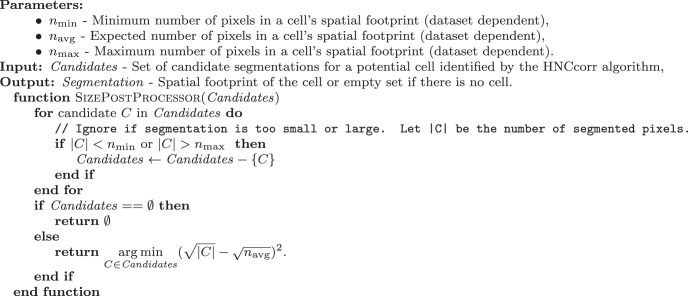
Size-based post-processor for the HNCcorr algorithm.

#### Effect of parameters in HNCcorr

Here we describe the effect of the parameters on the performance of HNCcorr in terms of cell detection quality and running time.

An important set of parameters for adapting HNCcorr to other datasets is a set that relates to the size of the cell. These parameters depend on the spatial resolution of the movie. These parameters are the patch size, the circle radius of the negative seeds, and the postprocessor parameters. The patch size *m* should be set such that cells containing the center of the patch are fully contained in the patch. The patch size *m* should not be set much larger, since it determines the size of the similarity graph and has a substantial impact on the running time. The radius *ρ* should be less than half the patch size, $\frac{m}{2}$. This ensures that the negative seeds fall inside the patch. The radius *ρ* should be chosen close to $\frac{m}{2}$, so a cell is contained in the circle. The radius *ρ* has no substantial impact on the running time or on the quality of HNCcorr, unless it is set too small and cells extend beyond the circle. The postprocessor parameters *n*_min_, *n*_max_ determine the size thresholds for the size of cell. If a segmentation falls outside this range, then it is discarded. If the range is set too small, then correct segmentations may be discarded. Similarly, the number of false positives (FP) may increase if the range is too large. The postprocessor parameters do not have a major impact on running time.

The seed selection parameters are *n*_grid_ and *p*_seed_. Recall that the parameter *n*_grid_ determines the resolution for partitioning the pixels into a grid, and the parameter *p*_seed_ determines the percentage of candidate seed pixels that are processed by HNCcorr. An increase in *n*_grid_ results in a cruder grid and in fewer positive seeds. An increase in *p*_seed_ directly increases the number of seeds that are processed. When the number of seeds is increased, then more cells may be detected. However, the number of FPs may increase as well. Also, the running time increases proportionally to the number of processed seeds.

The parameter *γ* determines the size of the reference set for (sim) ^2^. A smaller value of *γ* reduces the size of the feature vectors *R_i_*, and thus the computational cost. If *γ* is set too small, then the detection quality of HNCcorr may degrade since the reference set is no longer representative of the pixels in the patch. A *γ* value of ≥25% is sufficient for most datasets.

The parameter *n*_neg_ determines the number of negative seeds that are placed equidistant along the circle with radius *ρ*. This parameter has no impact on the running time or the detection quality when the circle is large enough to contain any cell located near the positive seed.

The sparse computation parameters *p* and *κ* determine the dimension of the low-dimensional space and the grid resolution in the low-dimensional space. The grid resolution *κ* determines the sparsity of the resulting graph. Higher values result in sparser graphs and lower running time. It was observed for general machine-learning problems that an increase in the grid resolution decreases the running time of the algorithms but has little effect on their accuracy (Hochbaum and Baumann, 2015). Similarly, the detection quality of HNCcorr is consistent for a wide range of grid resolutions, but running time decreases for larger *κ*. The dimension of *p* is set to a small value, such as 3 or 4, to balance computational cost with accuracy: on the one hand, we like the dimension *p* as low as possible to reduce the computation cost for sparse computation. On the other hand, higher values of *p* explain a larger fraction of the variance in the data and thus improve the accuracy of the projection.

The parameter *α* is the scale parameter for the Gaussian similarity. It has no effect on the running time of HNCcorr, but it could affect the quality of the similarities and hence also the detection quality of HNCcorr. If the segmentations produced by HNCcorr for new datasets are not satisfactory, then this parameter may need adjustment. It is possible to select a best performing value of *α*. This is done by visually comparing the segmentations produced by HNCcorr against a known set of cell footprints, for different values of *α*.

### Experimental evaluation on the Neurofinder public benchmark

To evaluate the experimental performance of HNCcorr, we it compare against other leading algorithms on the Neurofinder public benchmark. Specifically, we compare HNCcorr with the matrix factorization algorithms CNMF (Pnevmatikakis et al., 2016) and Suite2P (Pachitariu et al., 2016) as well as 3dCNN, a three-dimensional convolutional neuronal network. 3dCNN is an algorithm that only recently appeared on the Neurofinder benchmark. As of the time of submission of this article, there was no corresponding publication, nor was there a publicly available code for the algorithm. We requested the code for 3dCNN but have not received a response so far. We therefore are unable to make a comparison with 3dCNN except for the results posted on Neurofinder.

### Datasets

The Neurofinder community benchmark CodeNeuro (2016) is an initiative of the CodeNeuro collective of neuroscientists that encourages software tool development for neuroscience research. The collective also hosts the Spikefinder benchmark, which has led to improved algorithms for spike inference in calcium-imaging data (Berens et al., 2018). The Neurofinder benchmark aims to provide a collection of datasets with ground truth labels for benchmarking the performance of cell detection algorithms.

The benchmark consists of 28 motion-corrected calcium-imaging movies provided by four different laboratories. Datasets are annotated manually or based on anatomic markers. They differ in recording frequency, length of the movie, magnification, signal-to-noise ratio, and in the brain region that was recorded. The datasets are split into two groups: training datasets and test datasets. The 18 training datasets are provided together with reference annotations, whereas the reference annotations for the 9 test datasets are not disclosed. The test datasets and their undisclosed annotations are used by the Neurofinder benchmark to provide an unbiased evaluation of the performance of the algorithms. The characteristics of the test datasets are listed in Table 1. We note that some cells are not marked in the reference annotation. However, this does not invalidate the experimental analysis since the task of annotating cells remains equally difficult for each algorithm.

**Table 1:** Characteristics of the test datasets of the Neurofinder benchmark and their corresponding training datasets

Name	Source	Resolution	Length	Frequency	Brain region	Annotation method
00.00	Svoboda laboratory	512 × 512	438 s	7.00 Hz	vS1	Anatomical markers
00.01	Svoboda laboratory	512 × 512	458 s	7.00 Hz	vS1	Anatomical markers
01.00	Hausser laboratory	512 × 512	300 s	7.50 Hz	v1	Human labeling
01.01	Hausser laboratory	512 × 512	667 s	7.50 Hz	v1	Human labeling
02.00	Svoboda laboratory	512 × 512	1000 s	8.00 Hz	vS1	Human labeling
02.01	Svoboda laboratory	512 × 512	1000 s	8.00 Hz	vS1	Human labeling
03.00	Losonczy laboratory	498 × 467	300 s	7.50 Hz	dHPC CA1	Human labeling
04.00	Harvey laboratory	512 × 512	444 s	6.75 Hz	PPC	Human labeling
04.01	Harvey laboratory	512 × 512	1000 s	3.00 Hz	PPC	Human labeling

Data reproduced from Neurofinder (CodeNeuro, 2016).

All datasets can be downloaded directly from the Neurofinder benchmark for cell identification (CodeNeuro, 2016). The movies were provided to the algorithms without further preprocessing.

### Active versus inactive cells

We group the datasets into the following two subgroups: datasets in which most annotated cells are active (i.e., have a detectable fluorescence signal other than noise); and datasets in which most annotated cells are inactive. We make this distinction because inactive cells cannot be found by CNMF, Suite2P, and HNCcorr due to the lack of measurable changes in fluorescence other than noise. These cells are detectable with shape-based detection algorithms [S. Gao, (https://bit.ly/2UG7NEs)], when the brightness of the cell differs from the background.

**Figure 4.**
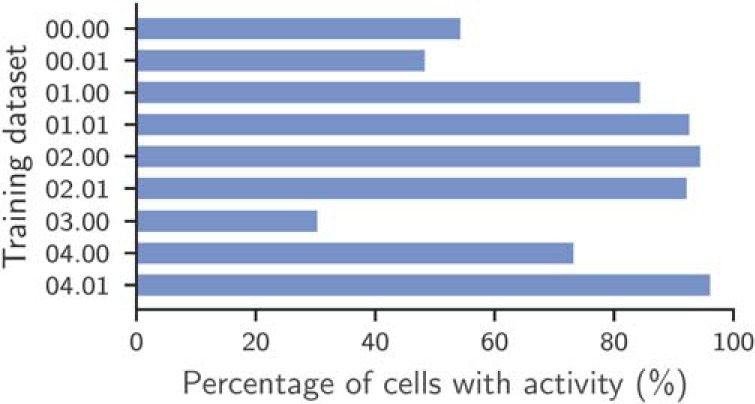
Approximate percentage of active cells among annotated cells in the training datasets. To determine the activity of the cells in a movie, we used the following approximate analysis. First, we downsample the movie by averaging 10 frames. For every annotated cell, we compute the average intensity over time of the pixels in the spatial footprint. This time series is used an estimate of the signal of the cell. A cell is then considered active if the Δ *f*/*f* (Jia et al., 2011) of this time series is at least 3.5 SDs above the median of Δ *f*/*f* for a minimum of 3 potentially nonsequential time steps. Due to the approximate nature of this analysis, its interpretation should be limited to understanding the general ratio between active and inactive cells in the datasets.

The datasets with inactive cells are as follows: 00.00, 00.01, and 03.00. The presence of inactive cells in these datasets has been noted in previous work (Pachitariu et al., 2016) as well as in a discussion (issues 16 and 24) of reference annotation of the benchmark on GitHub. Experimentally, we also observed that the percentage of annotated cells that are active is substantially lower for training datasets 00.00, 00.01, and 03.00, as shown in Figure 4. Results for the test datasets could not be evaluated due to the lack of a reference annotation, but similar results are expected.

### Evaluation metrics

The list of cells identified by each of the algorithms is compared with the reference annotation. For this comparison, we use the submissions on the Neurofinder website. Each algorithm was submitted by the authors of that algorithm. This ensures that the results are representative of the performance of the algorithm. Furthermore, the evaluation is fair since none of the authors had access to the reference annotation.

The algorithms are scored based on their ability to reproduce the reference annotation using standard metrics from machine learning. Each cell in the reference annotation is counted as a true positive (TP) if it also appears in the algorithm’s annotation. The cells in the reference annotation that are not identified by the algorithm are counted as false negatives (FNs), and the cells identified by the algorithm that do not appear in the reference annotation are counted as FPs. The performance of each algorithm is scored on a dataset based on $recall=\frac{TP}{\left(TP+FN\right)}$ and $precision=\frac{TP}{\left(TP+FP\right)}$. The overall performance of the algorithm on a dataset is summarized by the F1-score, which is the harmonic mean of recall and precision. For all metrics, higher scores are better.

A cell in the ground truth annotation is identified if the center of mass of the closest cell in the annotation of the algorithm is within 5 pixels. These two cells are then matched and can no longer be matched to another cell. Each cell in either annotation is thus matched at most once.

### Statistical analysis

Throughout this article, we apply descriptive analysis on a set of undisclosed test datasets. We assume for this analysis that the test datasets are drawn independent, identically distributed (i.i.d.) from the distribution of two-photon calcium-imaging datasets. The *p* values are inferred from a lookup table with quantiles for the Wilcoxon signed rank test.

### Code accessibility

The software used to generate the results in this work is available for noncommercial use at https://github.com/quic0/HNCcorr.

### Computational hardware

The experiments were run with MATLAB 2016a on a single core of a Linux machine running Linux Mint 18.1. The machine is equipped with an Intel i7-6700HQ CPU running at 2.60 GHz and 16GB RAM. The algorithm also runs on a standard desktop computer or a laptop.

### Algorithmic implementations for experimentation

**Figure 5.**
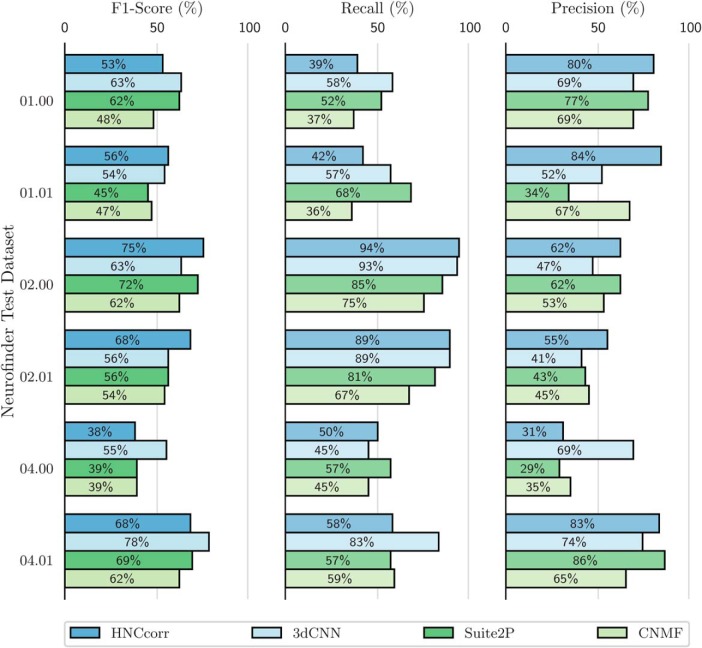
Cell identification scores for the HNCcorr, CNMF, and Suite2P algorithms on the Neurofinder test datasets with active cells. For each of the listed metrics, higher scores are better. The data are taken from Neurofinder submissions Sourcery by Suite2P, CNMF_PYTHON by CNMF, 3dCNN by ssz, and submission HNCcorr by HNCcorr.

The results in Figures 5  and 8 are taken directly from Neurofinder (CodeNeuro, 2016) and were produced by the authors of the respective algorithms. In the remainder, we describe the HNCcorr implementation used for all experiments including those reported in Figures 5  and 8. We also describe the CNMF and Suite2P implementation used for all experiments except for the results reported in Figures 5  and 8.

#### HNCcorr

All datasets for HNCcorr were preprocessed by averaging every ten frames into a single frame to reduce the noise. All parameters were kept at their default settings with the exception of dataset specific parameters listed in Table 2. These parameters are dataset dependent due to varying cell sizes across datasets. The parameters were tuned to maximize the F1-score on the associated training datasets.

**Table 2:** Dataset dependent parameter values used for the HNCcorr implementation. Parameters were selected to maximize the F1-score on the corresponding neurofinder training datasets

Dataset	Patch size	Radius circle	Size	Postprocessor		
		Negative seeds	Superpixel	Lower bound	Upper bound	Expected size
	(*m*)	(ρ)	(*k*)	(*n*_min)_	(*n*_max)_	(*n*_avg)_
00.00	31 × 31	10 pixels	5 × 5	40 pixels	150 pixels	60 pixels
00.01	31 × 31	10 pixels	5 × 5	40 pixels	150 pixels	65 pixels
01.00	41 × 41	14 pixels	5 × 5	40 pixels	380 pixels	170 pixels
01.01	41 × 41	14 pixels	5 × 5	40 pixels	380 pixels	170 pixels
02.00	31 × 31	10 pixels	1 × 1	40 pixels	200 pixels	80 pixels
02.01	31 × 31	10 pixels	1 × 1	40 pixels	200 pixels	80 pixels
03.00	41 × 41	14 pixels	5 × 5	40 pixels	300 pixels	120 pixels
04.00	31 × 31	10 pixels	3 × 3	50 pixels	190 pixels	90 pixels
04.01	41 × 41	14 pixels	3 × 3	50 pixels	370 pixels	140 pixels

For the experiments reported in Figures 5 and 8, we used non-default values for the reference set sampling rate (*γ* = 100%) and the grid resolution used for sparse computation (*κ* = 25). The values differ from the default since the results of these experiments were uploaded to the Neurofinder benchmark before we optimized the accuracy/running time trade-off. These changes should have a marginal effect on the cell detection quality of the algorithm, but they result in increased running times.

#### CNMF

The CNMF implementation was obtained from https://github.com/epnev/ca_source_extraction. The base configuration was taken from the file run_pipeline.m in the CNMF repository. We turned off the patch-based processing, and set the number of cells, as denoted by *K*, equal to 600 unless specified otherwise. We used the same values for the maximum and minimum cell size, max_size_thr and min_size_thr, as we used in HNCcorr. We also set the temporal down-sampling tsub to 10 to match the down-sampling used by the HNCcorr algorithm.

#### Suite2P

The Suite2P implementation was obtained from https://github.com/cortex-lab/Suite2P. The base configuration was taken from the file master_file_example.m in the Suite2P repository. The diameter parameter was tweaked per dataset to maximize the F1-score on the Neurofinder training datasets. The selected values per (dataset) are: 8 (00.00), 10 (00.01), 13 (01.00), 13 (01.01), 11 (02.00), 11 (02.01), 12 (03.00), 11 (04.00), and 12 (04.01).

## Results

### HNCcorr is a top performer on the Neurofinder benchmark for cell identification

We provide two comparisons of algorithm performance on the Neurofinder benchmark datasets. First, we analyze the performance of HNCcorr, CNMF, Suite2P, and 3dCNN on the Neurofinder test datasets with active cells. Second, we compare the top Neurofinder submissions across all test datasets.

### Active cell identification

The experimental performance of the algorithms HNCcorr, 3dCNN, CNMF, and Suite2P on the six test datasets containing active cells is shown in Figure 5 and Table 3. Overall, the HNCcorr algorithm has superior performance across datasets compared with the matrix factorization algorithms. The HNCcorr algorithm achieves 15% (*p* = 0.05) relative improvement compared with CNMF in terms of average F1-score across datasets. It also attains a minor improvement of 4% compared with Suite2P. However, it performs 3% worse than 3dCNN, which detects both active and inactive cells unlike HNCcorr, Suite2P, and CMNF.

**Table 3:** Summary of statistical results

	Data	Type of test	*p* value
Figure 5	Drawn i.i.d.	One-sided signed rank test	HNCcorr vs. 3dCNN: *p* > 0.4,
F1-score		(uncorrected for multiple tests)	HNCcorr vs. Suite2P: *p* > 0.2,
			HNCcorr vs. CNMF: *p* ≤ 0.05.
Figure 8	Drawn i.i.d.	One-sided signed rank test	HNCcorr + Conv2D vs. 3dCNN: *p* > 0.2,
F1-score		(uncorrected for multiple tests)	HNCcorr + Conv2D vs. Suite2P + Donuts: *p* > 0.1.
Figure 9	Drawn i.i.d.	One-sided signed rank test	HNCcorr vs. Suite2P: *p* > 0.4,
Solve time		(uncorrected for multiple tests)	HNCcorr vs. CNMF: *p* ≤ 0.10.

HNCcorr performs particularly well on datasets 02.00 and 02.01, where it attains substantially higher F1-scores than the other algorithms, due to higher detection capability, as measured by recall. Although 3dCNN is able to match the near-perfect recall of HNCcorr, it attains lower precision for datasets 02.00 and 02.01. This indicates that 3dCNN detects cells that either are not in the reference annotation or incorrectly mark noncell regions as cells.

**Figure 6.**
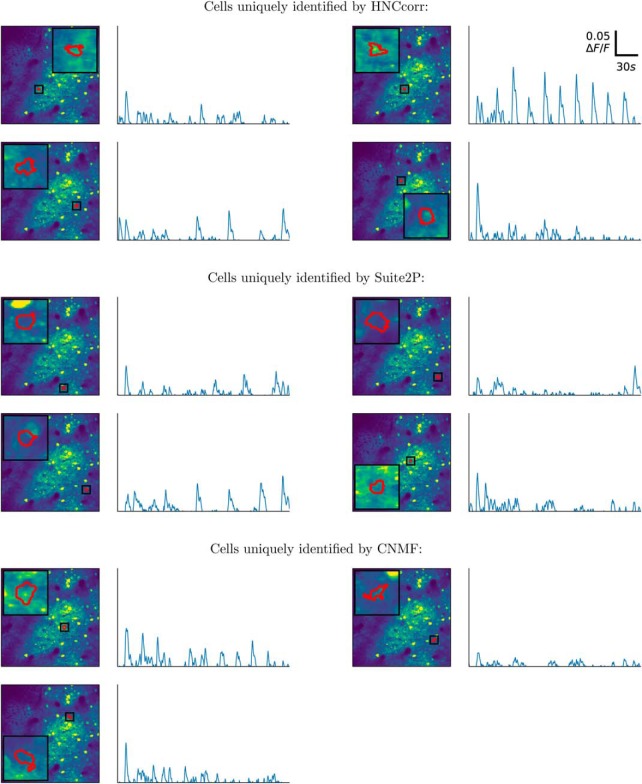
Footprints and ΔF/F signals for annotated cells in the Neurofinder training dataset 01.01 that were uniquely identified by one of the algorithms. Segmented footprints and Δ*F*/*F* signal for up to four cells are shown for each of the algorithms. Each cell also appeared in the reference annotation, but it was not identified by any of the other algorithms. The segmented spatial footprints are overlaid on the mean intensity image, scaled to show values from the first percentile up to the 99th percentile.

**Figure 7.**
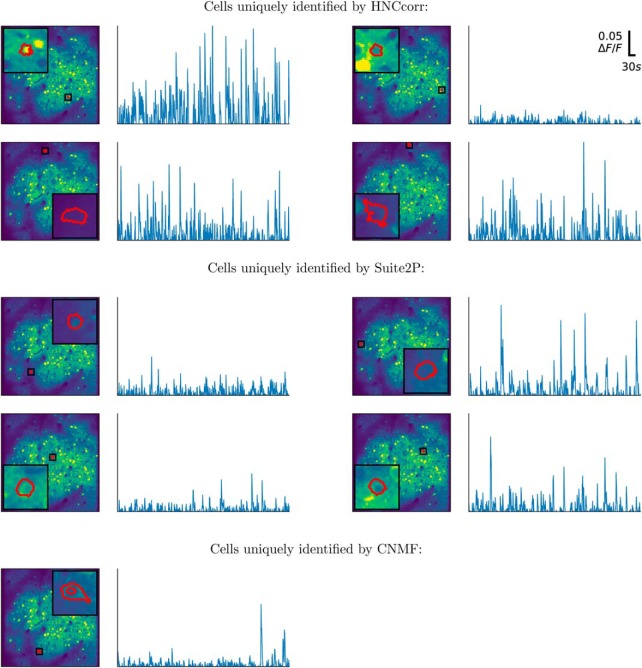
Footprints and Δ*F*/*F* signals for annotated cells in the Neurofinder training dataset 02.00 that were uniquely identified by one of the algorithms. Segmented footprints and Δ*F*/*F* signal for up to four cells are shown for each of the algorithms. Each cell also appeared in the reference annotation, but it was not identified by any of the other algorithms. The segmented spatial footprints are overlaid on the mean intensity image, scaled to show values from the first percentile up to the 99th percentile.

When HNCcorr, CNMF, and Suite2P are applied to the annotated, training datasets 01.01 and 02.00, each of the algorithms was able to uniquely detect some cells. These cells were not identified by the other algorithms, but were present in the reference annotation. Figures 6 and 7 show up to four examples of these cells for each of the algorithms. CNMF uniquely detected only three cells on the training dataset 01.01 and one cell for the 02.00 training dataset. HNCcorr thus finds meaningful cells that are not identified by the other algorithms.

### Leading Neurofinder submissions

**Figure 8.**
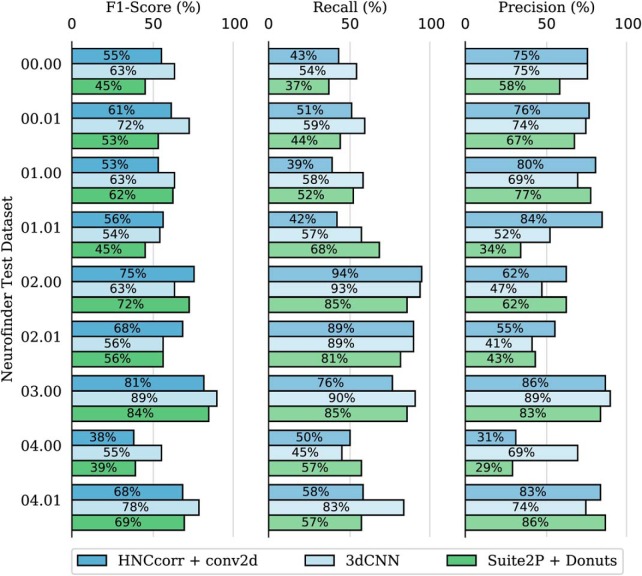
Cell identification scores on all test datasets for the three leading submissions of the Neurofinder benchmark in July 2018. For each of the listed metrics, higher scores are better. The 3dCNN entry is based on the Neurofinder submission 3dCNN by ssz. The Suite2P + Donuts (Pachitariu et al., 2013) entry is taken from the submission Sourcery by Suite2P. It uses the Donuts algorithm for datasets 00.00, 00.01, and 03.00 and the Suite2P algorithm for the remaining datasets. The HNCcorr + Conv2d entry is taken from the submission HNCcorr + conv2d by HNCcorr. It uses the Conv2d algorithm [S. Gao, (https://bit.ly/2UG7NEs)] for datasets 00.00, 00.01, and 03.00 and the HNCcorr algorithm for the remaining datasets. The results obtained with the Conv2d algorithm reported here differ slightly from the Conv2d submission on the Neurofinder benchmark since the Conv2d model was retrained by the authors of this article.

HNCcorr and matrix factorization algorithms identify cells based on their unique fluorescence signal. As such, they are able to detect cells that activate (i.e. have one or more spikes in calcium concentration), but they cannot detect cells without any signal. As discussed, Neurofinder datasets 00.00, 00.01, and 03.00 have a large number of inactive cells. Therefore, matrix factorization algorithms and HNCcorr detect only a small percentage of cells in these datasets. The leading Neurofinder submission for both Suite2P and HNCcorr therefore relies on a shape-based detection algorithm for these datasets. The HNCcorr + Conv2d submission uses the Conv2d [S. Gao, (https://bit.ly/2UG7NEs)] neural network for datasets 00.00, 00.01, and 03.00, and HNCcorr for the remaining datasets. Similarly, Suite2P + Donuts submission uses Donuts (Pachitariu et al., 2013) for the datasets 00.00, 00.01, and 03.00, and Suite2P for the remaining datasets. Together with the 3dCNN, these submissions are the top three submissions for the Neurofinder benchmark. Figure 8 and Table 3 shows how the three submissions compare. In particular, the 3dCNN algorithm outperforms the other shape-based detection algorithms on datasets 00.00, 00.01, and 03.00. As discussed before, HNCcorr attains higher F1-scores for the 02.00 and 02.01 datasets.

### HNC improves over spectral clustering

HNCcorr uses the clustering model HNC to identify the spatial footprint of a cell. Alternatively, one could use the spectral clustering method. To evaluate the impact of HNC, we tested a variant of HNCcorr where we replaced HNC with spectral clustering. Similarly, we also evaluated the effect of the (sim) ^2^ similarity measure by substituting the (sim) ^2^ in HNCcorr with the correlation similarity measure.

For spectral clustering, we consider the following two commonly used approaches for postprocessing of the eigenvector(s): The “threshold” method by Shi and Malik (2000) and the “*k*-means” method for *k* = 2 by Ng et al. (2002). The threshold method requires as input the number of pixels in a cluster, which we set to 80 pixels, the expected cell size used for postprocessing in HNCcorr. (We apply spectral clustering on a complete graph since this provides slight improvement in F1-score compared with the use of sparse computation.)

We compared the approaches on the Neurofinder 02.00 training dataset while retaining the same seed selection and postprocessing methods as used by HNCcorr. In Table 4, we provide the performance of the three clustering methods: HNC, spectral clustering (threshold), and spectral clustering (*k*-means). For each clustering method, we compare the use of correlation similarity to the use of (sim) ^2^. As seen in Table 4, HNC has a substantially higher F1-score than the two spectral clustering methods irrespective of the similarity measure used. This is primarily due to an increase in the precision score. For each clustering method, we observe that (sim) ^2^ provides a slight improvement over the use of correlation as a similarity measure. The results indicate that the choice of HNC as a clustering method is primarily responsible for the improved performance of HNCcorr.

**Table 4:** F1, Precision, and recall scores for three different clustering methods on the neurofinder 02.00 training dataset with the same seed selection and postprocessing methods as HNCcorr

	Correlation similarity			(sim) ^2^ similarity		
Clustering model	F1	Precision	Recall	F1	Precision	Recall
HNC	70.3	65.6	75.6	73.8	72.6	75.1
Spectral clustering (*k*-means)	46.5	41.9	52.3	49.2	49.2	49.2
Spectral clustering (threshold)	22.4	13.1	77.2	23.7	14.4	66.0

For each clustering method, results are shown with two similarities measures: correlation and (SIM)^2^.

### HNCcorr is at least as fast as matrix factorization algorithms

**Figure 9.**
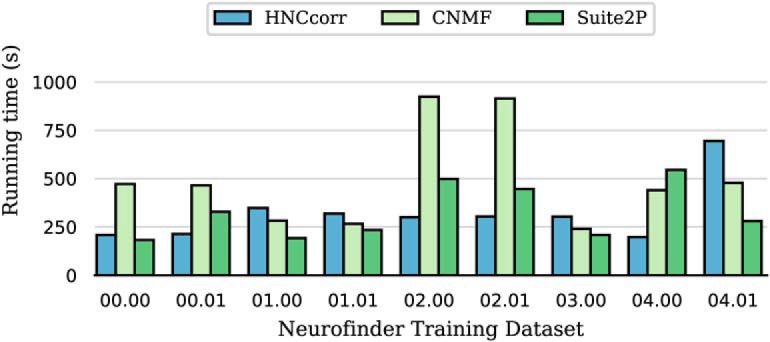
Running time results for nine training datasets of the Neurofinder benchmarks. Running times are based on a single evaluation.

We compared the running time performance of HNCcorr, CNMF, and Suite2P on nine training datasets of the Neurofinder benchmark. 3dCNN was excluded since the underlying code is not available. Running time results are given in Figure 9 and Table 3. On average, HNCcorr is 1.5 times faster than CNMF. Compared with Suite2P, HNCcorr performs equally well on average. We observed similar performance for a large experimental dataset consisting of 50,000 frames not reported here.

The running time of HNCcorr is dominated by the computation of the (sim ^2^) similarity weights and sparse computation. The running time of the algorithm scales approximately linearly in the number of edges in the graph and the number of pixels in the patch. Sparse computation helps to control the cost of computing the pairwise similarities by controlling the sparsity of the similarity graph *G*, which is determined by the grid resolution *κ* in sparse computation. A higher grid resolution results in a sparser graph.

### Performance of CNMF strongly depends on the user-specified number of cells

Currently, the most commonly used algorithm is the matrix factorization algorithm CNMF. We briefly sketch the idea behind matrix factorization algorithms. These algorithms rely on the data-generating process for calcium-imaging data *F* = *A C* + *B* + i.i.d. noise, where *F* is a known matrix containing the recorded data of the intensity of each pixel over time, matrix *A* contains the spatial contribution of each cell to each pixel, matrix *C* captures the intensity of each cell over time, and matrix *B* contains a time-varying background signal. The problem is then to infer *A*, *C*, and *B* given the matrix *F*. In general, the matrices are inferred by minimizing the objective function $\left||F-AC-B|{|}^{2}+\Omega (A,B,C\right)$ subject to non-negativity constraints, where ||·|| is a matrix norm and Ω(A,B,C) is a regularization function on the matrices *A*, *B*, and/or *C*. All matrix factorization algorithms (Mukamel et al., 2009; Pnevmatikakis and Paninski, 2013; Pnevmatikakis et al., 2013a, 2016; Diego-Andilla and Hamprecht, 2014; Maruyama et al., 2014; Pachitariu et al., 2016; Levin-Schwartz et al., 2017) present variants of this approach.

The matrix factorization model assumes that the number of cells (components) is provided in advance. This parameter is necessary to determine the sizes of matrices *A* and *C*. This number is either user specified or generated with the use of heuristics. A typical heuristic works by preselecting the number of components and merging components with sufficiently similar signals throughout the algorithm. In particular, the CNMF algorithm requires an input parameter *K* that determines the initial number of components, cells, and then merges components with sufficiently similar signals heuristically. We observe for CNMF that the choice of value for the parameter *K* is critical to the performance of the CNMF algorithm. This makes the algorithm difficult to use in practice since one cannot easily determine the appropriate value for the number of signal components *K*.

**Figure 10.**
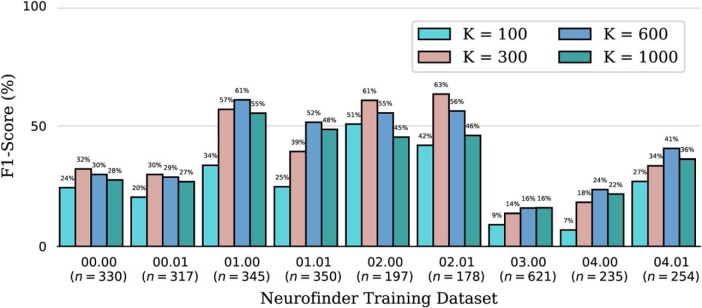
F1-score for the CNMF algorithm for various values of *K* on nine training datasets of the Neurofinder benchmark. The parameter *K* specifies the number of cells to consider initially for the matrix factorization. The number *n*, reported in parenthesis, is the number of cells in the reference annotation of the dataset.

We ran the CNMF algorithm for different values of *K* on nine different training datasets of the Neurofinder benchmark. For each of the datasets, we report in Figure 10 the F1-score obtained by the CNMF algorithm with the number of cells *K* set to 100, 300, 600, or 1000. The F1-score is obtained by comparing the cells found with the reference annotation. For most datasets, the F1-score attained by the algorithm depends strongly on the value of *K*. For example, the F1-score for the Neurofinder 02.01 training dataset is 42% for *K* = 100, 63% for *K* = 300, and 46% for *K* = 1000. This shows that setting *K* either too low or too high can negatively affect performance. This makes the parameter selection of *K* a difficult process.

Even knowing the true number of cells is insufficient, as seen in Figure 10, for, for example, dataset 04.01. This dataset has 254 cells, but CNMF with *K* = 300 provides a lower F1-score than with *K* = 600. In most cases, it is preferable to overestimate the number of cells, but by how much depends on the dataset.

**Figure 11.**
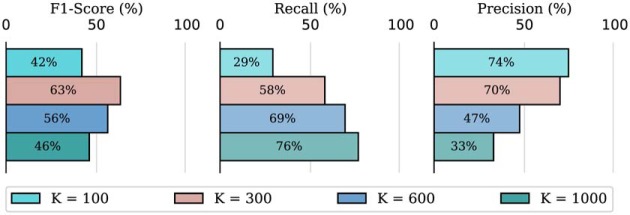
Cell identification scores for the CNMF algorithm for various values of *K* on the Neurofinder 02.01 training dataset. The parameter *K* specifies the number of cells to consider initially for the matrix factorization. This dataset has 178 cells in its reference annotation.

Figure 10 reports for each dataset the F1-score for various values of *K*. To provide additional insight into the effect of the choice of the parameter *K*, we report also the precision and recall for the Neurofinder 02.01 training dataset for various values of *K* in Figure 11. We conclude that the change in F1-score results from a trade-off between recall and precision. When *K* increases, recall improves but precision decreases.

Note that HNCcorr does not require an estimate of the total number of cells. Instead, it returns all segmentations that were accepted in postprocessing. This removes the need for parameter tuning of the number of cells *K* and guarantees consistent results.

## Discussion

### Local optimization in matrix factorization algorithms versus global optimization in HNCcorr

Non-negative matrix factorization is a powerful model for cell detection in calcium imaging. It is used by algorithms such as CNMF and Suite2P. The strength of the model is its ability to discern the signals of overlapping cells, which is particularly valuable in calcium-imaging datasets recorded with one-photon microscopy. However, the model is difficult to solve since the problem is nonconvex (Lee and Seung, 2001) and intractable (NP-hard) (Vavasis, 2009). Hence, the algorithms used for matrix factorization problems only obtain locally optimal solutions. These solutions can be arbitrarily bad when compared with the best possible solution, the global optimum. As a consequence, cells may remain undetected by the algorithm because a poor local optimum was obtained. However, the user cannot determine whether this occurred from the output of the algorithm.

In contrast, the HNC optimization model used to segment cells in HNCcorr is guaranteed to find the best possible solution for the model. That is, the HNC model is solved to global optimality. This removes any dependence on solution techniques, and it uniquely maps the input graph to the resulting segmentations. This simplifies the process of diagnosing potential mistakes in the preprocessing, since there is a transparent mapping between model input and output.

### Toward a real-time implementation

The future for calcium imaging in neuroscience is in real-time data collection, enabling direct feedback experimentation and a myriad of applications. This requires fast and parallelized cell identification algorithms that work with streaming data. Parallelization is inherent to the design of HNCcorr since it considers cells independently. This enables a direct parallel implementation by considering multiple cells concurrently. The algorithm also has a low memory requirement since the HNCcorr algorithm only uses the data for a small patch of the movie for each cell.

### Beyond two-photon calcium imaging

Although the performance of HNCcorr is demonstrated here for two-photon calcium imaging, we anticipate that HNCcorr may well be effective for movies collected with other calcium-imaging techniques, such as one-photon and light-field calcium imaging (Prevedel et al., 2014). Movies collected with one-photon calcium imaging are characterized by large, blurry background fluctuations from cells outside the focal plane (Zhou et al., 2018). Similarly, cells in light-field calcium imaging have blurry spatial footprints due to an imperfect reconstruction of the three-dimensional space. These data features require the algorithm to be able to separate the signals of overlapping cells. Currently, the HNC model in HNCcorr does not explicitly capture this objective. Nevertheless, the (sim) ^2^ similarity measure should be able to capture the individual signals of the cells, since the signal of each cell will be unique to the pixels in its spatial footprint. The cells in the footprint will thus have a unique component in their correlation vectors, resulting in higher similarity between these pixels. Initial experimentation suggests that HNCcorr is able to detect cells for both types of movies.

10.1523/ENEURO.0304-18.2019.ed1 Extended Data 1 The MATLAB code used to generate the results reported in this work. Instructions are provided in the readme.md file. The code requires the JSONlab toolbox, the Imagesci toolbox, and a C-compiler for MATLAB. Download Extended Data 1, ZIP file.

## Synthesis

Reviewing Editor: Gustavo Deco, Universitat Pompeu Fabra Decisions are customarily a result of the Reviewing Editor and the peer reviewers coming together and discussing their recommendations until a consensus is reached. When revisions are invited, a fact-based synthesis statement explaining their decision and outlining what is needed to prepare a revision will be listed below. The following reviewer(s) agreed to reveal their identity: Anca Doloc-Mihu, Eva Dyer.

The manuscript proposes a new algorithm called HNCcorr for cell identification in Ca imaging movies. This algorithm is based on a new method called similarity squared that calculates the similarities between two pixels from the same cell (positive info) along with similarities between the background pixels (negative info) to find a global optimal solution more efficiently. Authors have tested their algorithm on the Neurofinder cell identification benchmark and it seems that the algorithm outperforms the existing algorithms in most of the testings. Sparse computation of the sim^2 weights speeds up the algorithm. The manuscript is well written, with good explanations on the details of the algorithm. The new algorithm is well tested against some of the top existing algorithms for Ca imaging task, and seems to outperform them in many ways. In my opinion, this algorithm, though far from being perfect, is a promising step forward towards a such imaging algorithm for the Ca imaging neuroscience field.I suggest acceptance with only minor editing corrections from the authors.

Authors used p-values from Wilcoxon Signed Ranks Test and performed their own statistical analysis of the results. Precision, recall and F1 scores were provided for analyzing the results.

I have downloaded the code. Authors provided clear instructions included in the file README.md on how to install and run their code. One needs to install several softwares to be able to do so: Matlab, jsonlab, C-compiler, imagesci toolbox for H5-files. Maybe this info should also appear briefly within the manuscript. A link to your Github code should also appear in the manuscript.

The proposed algorithm has the advantage of using positive along with negative existing pixel information to learn faster the task at hand. I believe this is a great observation. The algorithm is a step forward for Ca imaging analysis. The manuscript is well written, but I have the following minor suggestions for improving it further:

1) HNC in the Abstract needs to be explained/expanded such that reader can understand the acronym easily.

2) in Introduction, citation KliRosEic17 seems to not exist?

3) throughout the entire paper the correct citation for many authors is FirstAuthor et al., year. Sometimes you also provide 2nd author and sometimes not. Please correct.

4) in Intro the phrase “major advantage of 46 HNCcorr compared to most alternative algorithms is that the HNC optimization model used to identify cells 47 is solved efficiently (in polynomial time) to global optimally” does not sound right. Did you mean to global optimality?

5) Also following phrase at line 55 “This is a well-established mathematical framework for image segmentation, where nodes in the graph correspond to pixels, edges represent similarities between pairs of pixels, and an objective function that assigns a cost to any possible segmentation of the graph” seems incorrect.

6) In Figure 1, is better to not send reader to Figure 3 to see the patch. Instead, just show it here before A.

7) line 114: “The radius of the circle is chosen such that the any cell that contains the positive seed is guaranteed to be in the circle”. This is better if also illustrated within a graphics.

8) Line 132: “The method rst projects these feature vectors onto a low-dimensional space of dimension p using a fast 132 approximation of principal component analysis” You need to provide a reference to this approx of PCA or explain it in more detail.

9)line 198 “is determined”

10)I like Figure 3. Can you make the images a bit bigger though?

11) What happens if the sample for the reference set is smaller than 1/4th? When is 1/6th? or 1/10th?

-------------

Rev 2:

This manuscript provides a graph partitioning-based solution, HNCcorr, to identify cells in calcium imaging movies. The algorithm is a relatively straightforward extension of an existing method (HNC). While HNCcorr is shown to be competitive with and faster than existing SOTA methods, the results are somewhat incremental (over HNC). To better understand how this approach will impact the field, the authors must provide a clearer explanation and evaluation to show how their method differs from other graph-partitioning methods (like graph cuts).

Calcium imaging is now a standard method in neuroscience for recording large-scale neural activity at a cellular level in vivo, but the resulting datasets need automated methods to identify cells and extract their time-varying activity. This paper introduces a new graph-partitioning algorithm called HNCcorr to identify cells in calcium imaging datasets. The cells are identified as coherent clusters that are distinguished from other pixels using a correlation-based similarity measure to define the graph used in HNC. The algorithm uses combinatorial optimization and guarantees a globally optimal solution in polynomial time, as well as minimal dependence on initialization techniques. The effectiveness of the algorithm has been demonstrated on the Neurofinder cell identification benchmark.

While there are good qualities about this paper and it does provide a competitive and efficient solution for finding cells, my overall impression was that the writing was excessively technical and lacked necessary insights into why certain decisions were made throughout the development of the method. To be accessible to the broad readership of eNeuro, the tone of the paper and clarity should be significantly improved and matched to the readership.

In particular, it would be beneficial to introduce the reader to general graph-based methods for clustering and graph cuts and later compare HNC to standard methods for spectral clustering. The paper would also benefit from a more careful comparison of graph partitioning with spectral clustering or graph cuts and HNC, as well as a comparison across methods used to form the affinity matrix used to segment the dataset (e.g., correlation-based, the reference set based method (used HNCcorr), and other kernel metrics like radial basis functions).

Detailed Comments:

- HNC is used as an acronym form without having been introduced before (or anywhere else) and is also slightly confusing due to the lack of differentiating “HNC” from the rest of the sentence. (line 8)

- Perhaps simply saying that current state-of-the-art methods use non-convex optimization techniques would be more effective in getting the authors' point across?(line 20)

- Replace “optimally” with “optimality” (line 48)

- Replace technique with techniques or affect with affects (lines 53-54)

- How is the threshold for the “highest average correlation” determined while generating positive seeds (mentioned to be the top 40% of correlations, in lines 256-257)? How long do they take to be generated? (lines 108-109)

- How is the radius of the circle to generate negative seeds determined? Are there any other constraints on it other than being large enough to contain the positive seed? (lines 113-114)

- What are the features R_i? Unclear at this point. (Line 131)

- When measuring the correlation between two pixels, how long is the time window over which the correlation is computed?

- What are the cell-size ranges being used?

- Would changing the size range change the speed of the algorithm's running time? (line 221)

- Is the number 0.25 (fraction to be sampled for (SIM)2) obtained by trial-and-error, chosen arbitrarily, or is there a particular reason for choosing the same? (line 238)

What about using a more standard graph partitioning method like spectral clustering? Would be useful to see the advantage of HNC over graph cuts to understand whether the performance is linked to the definition of the graph or the HNC method.

- For the sparse computation step, the features of each pixel are projected into a lower dimensional space with PCA. How does the method depend on this step? How do you select the lower dimension to project to?

- On line 201, the authors state that “ Unlike the Normalized Cut problem, there is a very efficient algorithm available to solve the HNC problem”. >> However, normalized cuts requires computing an eigenvalue decomposition of the affinity matrix and this can be computed very efficiently. The authors should compare with graph cuts (or other spectral clustering methods) to demonstrate the advantages of HNC.

- This method appears to be highly dependent on the choice of seed pixels - the authors should provide an analysis of the robustness and sensitivity of their method to the choice of seeds.

- For timing comparisons, the authors of CNMF have published an online version of their method. The authors should compare their HNC approach to online matrix factorization (onACID).

- On line 292, the authors state that “ We make this distinction because inactive cells cannot be found by CNMF, Suite2P, and HNCcorr since all three algorithms rely on a characteristic cell signal for detection.” >> What does this mean exactly? What is a characteristic cell signal?
